# Cross-cultural adaptation and translation of the Pediatric Intensive Care Unit-Quality of Dying and Death into Brazilian Portuguese

**DOI:** 10.5935/0103-507X.20210086

**Published:** 2021

**Authors:** Daiane Ferreira da Silva, Carlos Eduardo Paiva, Bianca Sakamoto Ribeiro Paiva

**Affiliations:** 1 Research Group on Palliative Care and Health- Related Quality of Life, Hospital de Câncer de Barretos - Barretos (SP), Brazil.; 2 Department of Clinical Oncology, Breast and Gynecology Division, Hospital de Câncer de Barretos - Barretos (SP), Brazil.

**Keywords:** Attitude to death, Translations, Surveys and questionnaires, Child, Intensive care units, pediatric

## Abstract

**Objectives:**

To translate and culturally adapt the Pediatric Intensive Care Unit-Quality of Dying and Death questionnaire into Brazilian Portuguese.

**Methods:**

This was a cross-cultural adaptation process including conceptual, cultural, and semantic equivalence steps comprising three stages. Stage 1 involved authorization to perform the translation and cultural adaptation. Stage 2 entailed independent translation from English into Brazilian Portuguese, a synthesis of the translation, back-translation, and an expert panel. Stage 3 involved a pretest conducted with family caregivers and a multidisciplinary team.

**Results:**

The evaluation by the expert panel resulted in an average agreement of 0.8 in relation to semantic, cultural, and conceptual equivalence. The pretests of both versions of the questionnaire showed that the participants had adequate comprehension regarding the ease of understanding the items and response options.

**Conclusion:**

After going through the process of translation and cultural adaptation, the Pediatric Intensive Care Unit-Quality of Dying and Death caregiver and multidisciplinary team versions were considered culturally adapted, with both groups having a good understanding of the items. The questionnaires include relevant items to evaluate the process of death and dying in the intensive care setting, and suggest changes in care centered on patients and especially family caregivers, given the finitude of their children.

## INTRODUCTION

Death is the final stage of the natural life cycle, a time that may be surrounded by stigma, fear and anguish, especially by those who deny this vital stage. The perception of this process can be influenced by culture, beliefs, and society.^(^1^)^ In pediatrics, death is seen as an unnatural process and the sudden rupture of a child’s natural life cycle, which can be marked by moments of great physical and emotional pain for both the child and the family, as well as for professionals working in intensive care.^(^2^,^3^)^

Studies conducted in intensive care units (ICUs) in the US indicate that most pediatric patients will die in the intensive care setting, in which invasive measures and technological support are provided to patients and maintained even in the face of the irreversible prognosis of their disease.^(^4^)^ In Brazil, due to the increase in technologies added over the decades to improve intensive care for the pediatric population, the mortality rate in pediatric intensive care units (PICUs) can reach a maximum of 10%.^(^5^)^

The care offered at the end of a child’s life in the PICU should be focused on three main actors: the child, his/her caregivers, and the health professionals involved. Comfort measures should also be offered to the child to ease his/her suffering.

Faced with the inevitability of their child’s death, parents wish to have their decisions, values, and beliefs respected and shared. They believe in the importance of clear, sincere communication about their child’s prognosis and that their parental power should be respected by all members of the care team, even in the face of their child’s finitude.^(^6^-^8^)^ Hales et al.^(^1^)^ defined quality of death (QoD) as the assessment in the last days of life and the moment of death, respecting the way this moment is prepared, faced and experienced by those, who have a known terminal illness.

To evaluate the quality of death and dying in pediatric patients in the PICU environment, the Pediatric Intensive Care Unit-Quality of Dying and Death (PICU-QoDD) questionnaire was adapted, as it is frequently used to assess the Quality Of Dying And Death (QoDD), a construct that is capable of objectively measuring QoD in adult patients.^(^9^,^10^)^ Based on the QoDD,^(^9^)^ with changes and adaptations to the pediatric context, the PICU-QoDD was formed into two versions with 10 domains, containing 22 questions for caregivers, and 14 questions for the multidisciplinary team.

A total of 94 deaths was analyzed over 12 months in two PICUs in the northeastern US to validate the latter through responses from 159 professionals working in the cited period.^(^10^)^ The questionnaires significantly correlate QoD with the assistance provided by the staff in the PICU, and evaluate care centered on the family in the face of the suffering caused during the patient’s stay in the unit, consisting of 10 major domains: communication; symptom control; continuity of care; intensive support; caregiver needs; parental presence; psychological care; grieving; religiousness; and privacy. This instrument encompasses care centered on pediatric patients and the family, and reports the vision of the team that provides care to these patients. The PICU-QoDD has adequate psychometric properties for nurses (Cronbach’s α = 0.891) and physicians (Cronbach’s α = 0.959); it can measure the QoDD of pediatric patients in intensive care, but there have been no translations or cultural adaptations of the instrument outside the original language (English).^(^10^)^

The objective of this study was to translate and culturally adapt the PICU-QoDD into Brazilian Portuguese.

## METHODS

This was a cross-cultural adaptation process including conceptual, cultural, and semantic equivalence steps.

### Procedures

International methodology was adopted for the translation and cultural adaptation of both versions (that of the multidisciplinary team and that of caregivers), including translation, a synthesis of the translations, back-translation, an expert panel, and a pretest according to the methodology proposed by Beaton et al.^(^11^)^ and Souza et al.^(^12^)^

The following flowchart outlines the translation and cultural adaptation process (Figure 1).

### Stages of the study

#### Stage I

Stage I involved receiving authorization from an author of the original questionnaire to perform the translation and cultural adaptation of the PICU-QoDD.

#### Stage II

Stage II entailed the translation and cultural adaptation of the original instrument. International methodology was used^(^11^)^ for the translation from English to Brazilian Portuguese, which was performed by two independent translators with no knowledge of the questionnaire and who were native Portuguese speakers and also fluent in English. The translations were coded as T1 and T2. Next, a synthesis of the translations was generated in which a consensus of the translated versions was obtained, resulting in the T12 version.

Next, back-translation of the Portuguese into English (the original language of the instrument) was performed by two native English speakers fluent in Brazilian Portuguese; the back-translations were coded as B1 and B2. The results were analyzed, and a consensus was reached between the researchers from Brazil and one of the researchers who developed the original instrument. The members of the expert committee received a specific document with the material of versions T12 and B12 and were instructed to evaluate each item of the scale according to the semantic, cultural, and conceptual equivalences.^(^13^)^

The agreement among five experts from the fields of psychology, medicine, and nursing with experience in pediatric oncology and in intensive care was evaluated, allowing for the independent evaluation of each item. The analysis of the data was performed both qualitatively and via an analysis of the scores of the specialists’ answers. To establish the representativeness of each item, a 4-point Likert-type scale with scores ranging from 1 to 4 was used. The content validity index was calculated considering responses with scores of 4 (representative item), 3 (item requires little change to be representative), 2 (large change needed to be significant), and 1 (unrepresentative). This index was calculated based on the sum of the equivalences divided by the total number of items. The items were considered equivalent when the mean content validity index across the items was greater than 0.8, indicating that items were adequate for measuring the objectives of the instrument.^(^12^,^14^)^ An expert committee asked questions and provided suggested revisions for the questionnaire, such as modifying or removing items that were not appropriate for Brazilian culture.^(^15^)^

#### Stage III

##### Pretest

Pretest data collection occurred from September 2018 to May 2019 (caregivers) and from November to December 2018 (the multidisciplinary team). The version was applied to a pretest sample, which consisted of a small group that fitted the profile of people for whom the questionnaire was intended: caregivers whose children had died in the PICU and a multidisciplinary team that took care of the child during his/her last three days of life.

For this pretest phase, according to Souza et al.^(^11^)^ and Beaton et al.^(^12^)^ methodology, a sample size of 10 to 40 participants, inserted into the context that the instrument aims to evaluate, was necessary. Thus, the pretest sample was planned for 6 to 10 caregivers and 30 professionals from the PICU team.

##### Caregiver pretest

Those who were older than 18 years of either sex, considered to be the patient’s main caregivers and present during the last days of the child’s life in the PICU, and who were willing to participate after receiving a phone call to talk about the last days of the child’s life were included. Those with psychiatric disorders reported in their medical records, with significant hearing loss, or who were unable to read, and indigenous caregivers who did not have the same culture as the general Brazilian population were excluded.

After agreeing to participate, the Brazilian validated version of the Patient Health Questionnaire-9 (PHQ9)^(^16^)^ was applied with the objective of screening for depressive symptoms and suicidal thoughts that would prevent caregivers from participating in the study. Please note that the PHQ9 is not a diagnostic tool, but it can help in screening patients with suspected depression. For this study, which involves the context of death, this assessment was important. The PHQ9 is composed of nine questions, with a 4-point Likert scale (0 to 3), totaling 27 points. Respondents were classified according to the final score as follows: 0 - 4, no depression; 5 - 9, mild depression; 15 - 19, moderate to severe depression; and > 20, severe depression. As one of the eligibility criteria, caregivers with a score ≥ 12 and/or a positive score for the last question (suicidal thoughts) were excluded from the study and did not respond to the PICU-QoDD; indigenous caregivers were also excluded.

Caregivers were contacted by telephone within 4 weeks to 6 months after the child had passed away. Then, participant randomization was carried out to determine whether the participant would perform the pretest by telephone without the PICU-QoDD questionnaire or through a hand-delivered questionnaire. The randomization consisted of whether the caregiver would fill out the questionnaire by hand, or if the interviewer would read it aloud to the caregiver over the phone.

Study data were collected and managed using REDCap electronic data capture tools hosted at *Hospital de Cancer de Barretos*.^(^17^)^

##### Multidisciplinary team pretest

For the PICU-QoDD multidisciplinary team version, professionals who worked in the PICU (physicians, nurses and nursing technicians, physiotherapists, psychologists, and social workers) were recruited; those who were over 18, had worked in the PICU of the institution for at least 4 months, and had provided care to the patient and his/her caregiver before death were included.

The PICU participating in this study is part of a reference hospital in Brazil in the treatment of childhood cancer, and has 6 intensive care beds with an annual average of 240 admissions of patients with a predominantly clinical profile.

##### Instruments for pretest data collection

The following pre-test data were used:

- Sociodemographic and clinical characteristics of the patients: age, state of origin, tumor type, and treatment phase.

- Sociodemographic characteristics of the caregivers: gender, civil status, and number of children.

- Sociodemographic characteristics of professionals on the multidisciplinary team: gender, marital status, number of children, time since graduation, and time in intensive care units.

- Pretest evaluation questionnaire (PICU-QoDD): application of a semistructured questionnaire with Likert-type answers, with the purpose of identifying and solving any problem, especially in relation to not understanding each item. The participants gave their opinions about the items, stated if they had any doubts or constraints, and made suggestions to adapt the questionnaire for both versions (i.e., caregivers and the multidisciplinary team). The instrument was prepared for this research with the objective of identifying participants’ understanding and doubts regarding PICU-QoDD items and responses.

Following completion of the consensus version developed by specialists and researchers, pretests for caregivers and the multidisciplinary team were administered, which were part of Stage III of the cultural adaptation process (i.e., translation, cultural adaptation, and content validity).

This research was approved by the Research Ethics Committee of the *Hospital de Cancer de Barretos* under protocol 1502/2017. Permission was granted by the author of the original instrument for the entire validation process in Brazil. All participants signed an informed consent form.

### Statistical analysis

Descriptive statistical analysis was performed for the sociodemographic data for the caregivers and the multidisciplinary team. A descriptive analysis was used to examine agreement among the expert committee members in relation to the scale items. Semantic, cultural, and conceptual equivalences were determined by calculating the content validity index.

Study data were managed using REDCap electronic data capture tools, hosted at *Hospital de Cancer de Barretos,*^(^17^)^ and analyzed using IBM Statistical Package for the Social Sciences (SPSS), version 21.

## RESULTS

### Translation and cross-cultural adaptation

The translations and back-translations followed the proposed methodologies, and the PICU-QoDD questionnaire required minimum adjustments to obtain a version that ensured language equivalence, as shown in table 1. Subsequently, all translation and back-translation versions were sent for evaluation by the author of the original version of the PICU-QoDD. After taking his thoughts into account, the expert committee process began.

For the caregiver version, questionnaire items 1-G, 1-N, 9-B, 10-B, 16, and 17 received scores of 2 or 3, and the judges made suggested modifications. No item received a score lower than 2 (Table 1). The other items received scores of 4.

For the multidisciplinary team version, questionnaire items 1-F, 1-M, 6-B, and 7-B received scores of 2 or 3, and the judges made suggested modifications. No item scored lower than 2 (Table 2).

### Caregiver pretest

In the pretest, 28 pediatric patients who died and 44 caregivers of these patients were eligible. Regarding caregivers, 38 (86%) were excluded because 7 (16%) with PHQ9 scores ≥ 12.5; five (11%) were associated with caregivers who had PHQ9^(^16^)^ scores ≥12 (for these cases, the criterion of not contacting the second caregiver from the same family was adopted when one screened positive for depression or suicide risk). Two (4%) were indigenous caregivers who did not have the same culture as the general Brazilian population. Three (7%) had a history of psychiatric disorders; 1 (2.6%) was associated with a caregiver with psychiatric disorders; 17 (39%) were unable to be reached by telephone; and 3 (7%) refused to participate. Thus, 6 (14%) pretests were performed.

All participants (four fathers and two mothers) in the pretest were married, with a diversified level of education (nine to 10 years of schooling; and four, higher education). There was greater understanding and less interview time in the group that received the version of the questionnaire at home and had it read to them.

In general, there was a good understanding of the questionnaire items and response options, and only one caregiver had a question, specifically about Item 1G: “Did you feel that the clinical team cared about your child as an individual?” The caregiver suggested that the word *indivíduo* (“individual”) be changed to *ser humano* (“human being”).

Interestingly, during the pretest with caregivers, they were extremely pleased to be contacted by the research team, whom they had become fond of during the care of their child; consequently, they felt comfortable during the interview.

**Table 1:** Equivalences of the Quality of Dying and Death Questionnaire in the Pediatric Intensive Care items (caregivers) performed by the expert committee

Item	Equivalences	Semantics	Conceptual	Cultural
14	1G. Did you feel that clinical staff cared about your child as an individual?	0.6	1	1
21	1N. Were hospital clergy or chaplains available the way that you wanted them to be?	0.8	1	1
55	9B. Discussion with you during rounds	0.8	1	1
62	10B. Discussion with you during rounds	0.8	1	1
73	16. While your child was in the ICU, did anyone talk to you about grief and bereavement support that might be available to your family?	0.8	0.8	1
75	17. While your child was in the ICU, were you given any written materials on grief and bereavement?	0.8	0.8	1

ICU - intensive care unit.

**Table 2:** Equivalences of the Quality of Dying and Death Questionnaire in the Pediatric Intensive Care items (the multidisciplinary team) performed by the expert committee

Item	Equivalences	Semantics	Cultural	Conceptual
10	1F. Staff showed that they cared about the child as an individual	0.8	1	1
17	1M. Hospital clergy or chaplains were available	0.6	1	0.8
56	6B. Discussion with the family during rounds	0.6	1	0.8
63	7B. Discussion with the family during rounds	0.8	1	0.8

Table 1S (Supplementary material) shows the final version of the caregiver PICU-QoDD (English and Brazilian Portuguese).

### Multidisciplinary team pretest

The pretest was administered to 30 participants: 8 (27%) nurses, 9 (30%) nursing technicians, 7 (23%) intensive care physicians, 3 (10%) physiotherapists, 2 (7%) social workers, and 1 (3%) psychologist. Those who predominated were female (27/90%), white (23/76%), married/in a stable relationship (21/70%), had 1 or 2 children (22/73%), and had more than 3 years of work experience in intensive care (17/57%).

The professionals had an adequate understanding of the PICU-QoDD items and response options, and highlighted the need for changes to the items provided in table 3.

Table 2S (Supplementary material) displays the final version of the PICU-QoDD (English and Brazilian Portuguese) for the multidisciplinary team.

Table 4 outlines the participants’ sociodemographic characteristics.

## DISCUSSION

For this study, translation and cultural adaptation of the PICU-QoDD was performed to produce two versions: one for caregivers, and one for the multidisciplinary team. The methodology was adopted according to Beaton et al.^(^11^)^ to maintain process quality for application in Brazil.

Beliefs, customs, and cultural styles were taken into account during the transcultural process of translation and adaptation to avoid failures that could be barriers to the use of the instrument, invalidating the construct for the reality of the country for which it is intended.^(^18^,^19^)^

Semantic, conceptual, and cultural equivalences, as well as pretests with the target populations, were fundamental to adapting the instrument to Brazilian culture,^(^11^,^14^)^ requiring minimal changes suggested by the expert panel and adequate understanding of both versions of the PICU-QoDD (for caregivers and the multidisciplinary team). The results of the pretest comprehension showed that the questionnaires were in agreement with the original instrument’s content.^(^10^)^

Regarding the construct, a patient’s QoDD, this patient population has unique characteristics where contact with caregivers is mostly performed by telephone or other means of communication, as there is no longer a bond of care with the family *in loco*. Brazil is a large country, and the *Hospital de Cancer de Barretos* serves patients from five regions (i.e., nationally). Although contact was not made with the caregivers for four weeks to six months after the death, we noted how difficult it was for them to address the subject, which in Brazil involves cultural issues and barriers, especially when it comes to the death of a child. When facing the death of their child, parents experience an unimaginably painful process and very distressing moments that can affect the entire family for a long time, even for life. Parents faced with the death of their child are confronted with feelings of loss and helplessness, and many fail to seek positive coping strategies to cope.^(^7^,^20^)^ Thus, it was possible to identify, in this study, that of the 44 family members eligible to participate, only 6 accepted. We believe this was mainly due to the grieving process that these family members may have been experiencing. However, we believe that for the process of content validity of the PICU-QoDD, the sample of 6 family members was sufficient for the adaptation of the questionnaire, which also provided an accurate assessment of the barriers in assessing QoD in pediatric oncology, leading to research questions that culminate in the planning of future studies that address this theme with family members at the time of the pediatric patient’s care process.

**Table 3:** Quality of Dying and Death Questionnaire in the Pediatric Intensive Care pretest (the multidisciplinary team)

Item	Participants	Comments	Suggestions	Change
1I; 1R; 4A a R	2 (6.6)	Replace the words “clinical and health team”	Multidisciplinary team	Multidisciplinary team
7	9 (30)	Doubts about the statement in question 6	Emphasize/underline the differences in the final part of statements 6 and 7.	Statement 6: We would like to know where communication occurred with the family. Thinking about the communication between the team and the family that you participated in or observed, how much of this communication occurred in: Statement 7: Now please think about all the communication between the team and the family during the last 3 days of the child’s life. To the best of your knowledge, how much of this communication occurred in:
12D	8 (24)	Out of scope	Outside of my routine, reality, work, practice	Out of range/reality/routine of my practice

* Brazil’s minimum wage. Results expressed as n (%).

**Table 4:** Sociodemographic characteristics of the multidisciplinary team and caregivers included in the pretest

Variable	Frequency	
	Multidisciplinary team	Caregivers
Sex		
Female	27 (90)	2 (33.3)
Male	3 (10)	4 (66.7)
Race		
White	23 (76.7)	4 (66.7)
Black	2 (6.6)	-
Brown	5 (16.7)	2 (33.3)
Marital status		
Single	6 (20)	-
Married/in a stable relationship	21 (70)	6 (100)
Divorced	3 (10)	-
Number of children		
None	7 (23.3)	-
1	11 (36.7)	2 (33.3)
2	11 (36.7)	4 (66.7)
3	1 (3.3)	-
Religion		
Catholic	17 (56.7)	3 (50)
Spiritist	6 (20)	-
Evangelical	5 (16.7)	2 (33.3)
Ignored	2 (6.6)	1 (16.7)
Family income (minimum wage)*		
1 - 3	15 (50)	2 (33.3)
3 - 6	7 (23.3)	1 (16.7)
> 6 wage	8 (26.7)	3 (50)
Time of formation (years)		
< 1	2 (6.6)	-
1 - 5	8 (26.7)	-
6 - 10	9 (30)	2 (33.4)
> 11	11 (36.7)	4 (66.6)
Specialization in intensive care		
Yes	12 (40)	
No	18 (60)	
Years of work experience in intensive care (years)		
< 1	6 (20)	
1 - 3	7 (23.3)	
> 3	17 (56.7)	

Results expressed as n (%).

The eligibility criteria defined for this study were important to carefully determine whether caregivers were emotionally prepared to respond safely and without emotional distress to the questionnaire. The use of the PHQ9^(^16^)^ was essential to identify caregivers who were potentially ineligible to participate due to emotional conditions. Studies correlate family changes related to the loss of children with high rates of depression, among other psychological illnesses, in which family dynamics change significantly. Therefore, using instruments that can track symptoms of depression or risk of suicide are essential; it is effective to exclude individuals with such issues from studies that refer to the moment of the loss of a loved one, as including them may exacerbate suffering.^(^21^)^

Finally, this study was restricted to a center in Brazil in a city located in the interior of the state of São Paulo. However, despite the great geographic expansion of the country, all five regions share the same language, and although there are some cultural variations, this is not a factor that compromises the generalization of the instrument to the Brazilian population as a whole. Another limitation was that caregivers were approached less than a year after the death of their loved one, which may have been a contributing factor to the small number of caregivers in the pretest.

## CONCLUSION

The two Pediatric Intensive Care Unit-Quality of Dying and Death versions (for caregivers and the multidisciplinary team) were culturally adapted, with a good understanding of the items by both groups of participants. The questionnaires include relevant items that evaluate the process of dying and death in the intensive care setting, and can facilitate changes in care centered on patients (and even family caregivers) in view of one’s suffering in relation to the end of life.

Future research is needed to evaluate other psychometric properties of the Pediatric Intensive Care Unit-Quality of Dying and Death versions used in this study. In addition, it may be feasible to review the number of items and their similarity; as such, the questionnaire could be transformed into a shorter version to facilitate its application in the daily routine of the pediatric intensive care unit.

## Disponibilidade dos dados

Os dados que dão suporte a este estudo foram apresentados no artigo e materiais suplementares.
