# Supplementary material for: Cross-cultural adaptation and translation of the Pediatric Intensive Care Unit-Quality of Dying and Death into Brazilian Portuguese
Source: Rev Bras Ter Intensiva. 2021 Oct-Dec;33(4):592–9. doi: 10.5935/0103-507X.20210086 (PMC8889588; doi:10.5935/0103-507X.20210086)
Supplement: Supplementary file 1 [file rbti-33-04-0592-suppl01.pdf]

Cross-cultural adaptation and translation of the Pediatric Intensive Care Unit-Quality of Dying and Death into Brazilian Portuguese

Adaptação transcultural para o português brasileiro do questionário Pediatric Intensive Care Unit-Quality of Dying and Death

Daiane Ferreira da Silva[1], Carlos Eduardo Paiva[1,2], Bianca Sakamoto Ribeiro Paiva[1]

Table 1S - Original instrument PICU-QODD and Brazilian version - Family Caregiver

ORIGINAL	BRAZILIAN VERSION
PICU-QODD<br>A Child's and his/her parent's experience at the end of life in pediatric intensive care.<br>A survey of Parents	*PICU-QODD:*<br>*Experiência de uma criança e de seus pais no fim de vida em terapia intensiva pediátrica.*<br>*Uma pesquisa com os pais*
Thank you for agreeing to do this survey. This survey is about experience that you and your child had during the last 3 days of your child's stay in the pediatric intensive care unit (ICU).<br>We are interested in your experiences because we want to improve the care received by children at the end of life and their family members.<br>Most of these questions are designed to be answered by a certain set of responses, for example "yes or no" or "never – sometimes – often – always".<br>At the end of survey, there are three questions with no list of possible responses, so you can answer them in any way you wish.<br>All your answers to these questions will be kept completely confidential. No one at the hospital will know what your answers are, so you can be honest about your experiences there. The survey will be identified only by a code number.<br>We know it is difficult to think about your child's last few days. If you would prefer not to answer a particular question, just skip that question. You can also end the survey altogether if you wish	*Obrigado por concordar em responder esta pesquisa. Esta pesquisa é sobre a experiência que você e seu filho tiveram durante os 3 últimos dias de internação dele na unidade de terapia intensiva (UTI) pediátrica.*<br>*Estamos interessados em suas experiências porque queremos melhorar os cuidados oferecidos às crianças no final de vida e aos membros de suas famílias.*<br>*A maioria destas perguntas foi desenvolvida para ser respondida por certo tipo de respostas, por exemplo, "sim ou não" ou "nunca – às vezes – com frequência – sempre".*<br>*No final da pesquisa, há três perguntas sem uma lista de respostas possíveis, assim você poderá respondê-las da maneira que quiser.*<br>*Todas suas respostas a estas perguntas serão mantidas completamente confidenciais. Ninguém no hospital saberá quais são suas respostas, assim, você pode ser honesto quanto às suas experiências no hospital. A pesquisa será identificada apenas por um código com número.*<br>*Sabemos que é difícil para você relembrar os últimos dias de vida de seu filho. Se você preferir não responder a uma pergunta em particular, apenas pule essa pergunta. Você pode até parar de responder à pesquisa, se assim o desejar.*
**Section A**<br>1. For this first section of the survey, the question has 2 parts. The first part asks you to think about<br>HOW OFTEN something happened while you and your child were in a pediatric ICU.<br>The second part asks for YOUR RATING<br>For example, for the first question, how often did you feel that your child was free of pain? Your response choices are "never, sometimes, often, or always". Circle the number that best represents your experience.<br>Then the second part of the question is "how would you rate this aspect of the experience you and your child had in the ICU?" The responses range from 0 - 10, where 0 is "terrible" and 10 is "almost ideal". We Realize that the death of a child is one of the most difficult situations a family can face.<br>But we believe that the care provided by ICU staff to you and your child can make a difficult experience better or worse. That is, if your child's death had to happen, what kind of care would you want for you and your child? How close was your actual experience to the ideal? Please rate each aspect of your experience by circling the appropriate number on the scale from 0 to 10. Below is an example	***Seção A***<br>*1. Para esta primeira parte da pesquisa, a pergunta é dividida em 2 partes. A primeira parte pede que você pense sobre:*<br>*COM QUE FREQUÊNCIA alguma coisa aconteceu enquanto você e seu filho estavam na UTI pediátrica (UTI)*<br>*A segunda parte pedirá SUA AVALIAÇÃO*<br>*Por exemplo, para a primeira pergunta, com que frequência você achou que seu filho não sentia nenhuma dor? Suas escolhas de resposta são "nunca, às vezes, frequentemente ou sempre". Circule o número que melhor representa sua experiência.*<br>*Em seguida, a segunda parte da pergunta é "como você avaliaria este aspecto da experiência que você e seu filho tiveram na UTI?" As respostas variam de 0 - 10, onde 0 é "terrível" e 10 é "quase ideal". Nós sabemos que a morte de um filho é uma das mais difíceis situações que uma família pode enfrentar.*<br>*Mas, nós acreditamos que os cuidados prestados pela equipe da UTI a você e seu filho pode transformar uma experiência difícil em algo tolerável. Ou seja, se a morte de seu filho era inevitável, que tipo de cuidado você gostaria de ter tanto para você quanto para seu filho? A experiência que você passou, chegou perto de ser a experiência toleravél? Por favor, avalie cada aspecto de sua experiência fazendo um círculo no número apropriado na escala de 0 a 10. Veja o exemplo abaixo.*
Example:<br>While in the ICU during the last three days of your child's life, HOW OFTEN…<br><br>a. Did you feel that your child was free of pain?<br>Never 1 — Sometimes 2 — Often 3 — Always 4	*Exemplo:*<br>*Quando você estava na UTI durante os últimos três dias de vida de seu filho (a), COM QUE FREQUÊNCIA...*<br>*a. Você achou que seu filho não sentia nenhuma dor?*<br>*Nunca 1 — Às vezes 2 — Frequentemente 3 — Sempre 4*
Example:<br>How would you RATE this aspect of the experience you and your child had in the ICU?<br>Terrible — Almost ideal<br>1 2 3 4 5 6 7 8 9 10	*Exemplo:*<br>*Como você AVALIARIA este aspecto da experiência que você e seu filho (a) tiveram na UTI?*<br>*Terrível — Quase ideal*<br>*1 2 3 4 5 6 7 8 9 10*

Continue...

...continuation

ORIGINAL	BRAZILIAN VERSION
**PLEASE START THE SURVEY HERE:**	**POR FAVOR, COMECE A PESQUISA AQUI:**
While in the ICU during the last three days of your child's life, HOW OFTEN...	*Quando você estava na UTI, durante os últimos três dias de vida de seu filho (a), COM QUE FREQUÊNCIA...*
a. Did you feel that your child was free of pain?	*a. Você sentiu que seu filho(a) estava livre de dor?*
Never Sometimes Often Always	*Nunca Às vezes Frequentemente Sempre*
1 2 3 4	*1 2 3 4*
How would you RATE this aspect of the experience you and your child had in the ICU?	*Como você AVALIARIA este aspecto da experiência que você e seu filho(a) tiveram na UTI?*
Terrible Almost ideal	*Terrível Quase ideal*
1 2 3 4 5 6 7 8 9 10	*1 2 3 4 5 6 7 8 9 10*
b. Did you feel that your child was free of other troubling symptoms?	*b. Você sentiu que seu filho (a) estava livre de outros sintomas preocupantes?*
Never Sometimes Often Always	*Nunca Às vezes Frequentemente Sempre*
1 2 3 4	*1 2 3 4*
Terrible Almost ideal	*Terrível Quase ideal*
1 2 3 4 5 6 7 8 9 10	*1 2 3 4 5 6 7 8 9 10*
c. Did you feel that clinical staff responded quickly to your concerns about your child's symptoms?	*c. Você sentiu que a equipe clínica respondeu rapidamente às suas preocupações quanto aos sintomas do seu filho (a)?*
Never Sometimes Often Always	*Nunca Às vezes Frequentemente Sempre*
1 2 3 4	*1 2 3 4*
How would you RATE this aspect of the experience you and your child had in the ICU?	*Como você AVALIARIA este aspecto da experiência que você e seu filho (a) tiveram na UTI?*
Terrible Almost ideal	*Terrível Quase ideal*
1 2 3 4 5 6 7 8 9 10	*1 2 3 4 5 6 7 8 9 10*
d. Did you feel that clinical staff gave you information about your child in a way that you could understand?	*d. Você sentiu que a equipe clínica forneceu informações sobre seu filho (a) de uma maneira que você pudesse entender?*
Never Sometimes Often Always	*Nunca Às vezes Frequentemente Sempre*
1 2 3 4	*1 2 3 4*
How would you RATE this aspect of the experience you and your child had in the ICU?	*Como você AVALIARIA este aspecto da experiência que você e seu filho (a) tiveram na UTI?*
Terrible Almost ideal	*Terrível Quase ideal*
1 2 3 4 5 6 7 8 9 10	*1 2 3 4 5 6 7 8 9 10*
e. Did you feel comfortable asking clinical staff question?	*e. Você se sentiu à vontade para fazer perguntas à equipe clínica?*
Never Sometimes Often Always	*Nunca Às vezes Frequentemente Sempre*
1 2 3 4	*1 2 3 4*
How would you RATE this aspect of the experience you and your child had in the ICU?	*Como você AVALIARIA este aspecto da experiência que você e seu filho (a) tiveram na UTI?*
Terrible Almost ideal	*Terrível Quase ideal*
1 2 3 4 5 6 7 8 9 10	*1 2 3 4 5 6 7 8 9 10*
f. Did you feel that clinical staff kept you waiting when you had questions about your child?	*f. Você sentiu que a equipe clínica fez você esperar quando você perguntava sobre seu filho(a)?*
Never Sometimes Often Always	*Nunca Às vezes Frequentemente Sempre*
1 2 3 4	*1 2 3 4*
How would you RATE this aspect of the experience you and your child had in the ICU?	*Como você AVALIARIA este aspecto da experiência que você e seu filho tiveram na UTI?*
Terrible Almost ideal	*Terrível Quase ideal*
1 2 3 4 5 6 7 8 9 10	*1 2 3 4 5 6 7 8 9 10*
g. Did you feel that clinical staff cared about your child as an individual?	*g. Você sentiu que a equipe clínica se preocupou com seu filho como um indivíduo?*
Never Sometimes Often Always	*Nunca Às vezes Frequentemente Sempre*
1 2 3 4	*1 2 3 4*
How would you RATE this aspect of the experience you and your child had in the ICU?	*Como você AVALIARIA este aspecto da experiência que você e seu filho tiveram na UTI?*
Terrible Almost ideal	*Terrível Quase ideal*
1 2 3 4 5 6 7 8 9 10	*1 2 3 4 5 6 7 8 9 10*
h. Did you feel that clinical staff supported you emotionally?	*h. Você sentiu que a equipe clínica te apoiou emocionalmente?*
Never Sometimes Often Always	*Nunca Às vezes Frequentemente Sempre*
1 2 3 4	*1 2 3 4*
How would you RATE this aspect of the experience you and your child had in the ICU?	*Como você AVALIARIA este aspecto da experiência que você e seu filho tiveram na UTI?*
Terrible Almost ideal	*Terrível Quase ideal*
1 2 3 4 5 6 7 8 9 10	*1 2 3 4 5 6 7 8 9 10*
i. Did you feel that your family's wishes and decisions were respected by clinical staff?	*i. Você sentiu que os desejos e decisões da família foram respeitados pela equipe clínica?*
Never Sometimes Often Always	*Nunca Às vezes Frequentemente Sempre*
1 2 3 4	*1 2 3 4*
How would you RATE this aspect of the experience you and your child had in the ICU?	*Como você AVALIARIA este aspecto da experiência que você e seu filho tiveram na UTI?*
Terrible Almost ideal	*Terrível Quase ideal*
1 2 3 4 5 6 7 8 9 10	*1 2 3 4 5 6 7 8 9 10*

Continue...

...continuation

ORIGINAL	BRAZILIAN VERSION
j. Did you feel you had enough opportunities to discuss options about your child's care with the healthcare team?<br>Never Sometimes Often Always<br>1 2 3 4<br>How would you RATE this aspect of the experience you and your child had in the ICU?<br>Terrible Almost ideal<br>1 2 3 4 5 6 7 8 9 10	*j. Você sentiu que teve oportunidades suficientes para discutir as opções de tratamento de seu filho (a) com a equipe de cuidados de saúde?*<br>*Nunca Às vezes Frequentemente Sempre*<br>*1 2 3 4*<br>*Como você AVALIARIA este aspecto da experiência que você e seu filho tiveram na UTI?*<br>*Terrível Quase ideal*<br>*1 2 3 4 5 6 7 8 9 10*
K. Were these conflicts between you and the clinical staff about the best way to care for your child?<br>Never Sometimes Often Always<br>1 2 3 4<br>How would you RATE this aspect of the experience you and your child had in the ICU?<br>Terrible Almost ideal<br>1 2 3 4 5 6 7 8 9 10	*k. Existiram conflitos entre você e a equipe clínica sobre a melhor maneira de cuidar de seu filho(a)?*<br>*Nunca Às vezes Frequentemente Sempre*<br>*1 2 3 4*<br>*Como você AVALIARIA este aspecto da experiência que você e seu filho tiveram na UTI?*<br>*Terrível Quase ideal*<br>*1 2 3 4 5 6 7 8 9 10*
l. Did you find it difficult to meet your own basic physical needs (accessible bathroom, showers, affordable meals, places to stay, parking, etc..)?<br>Never Sometimes Often Always<br>1 2 3 4<br>How would you RATE this aspect of the experience you and your child had in the ICU?<br>Terrible Almost ideal<br>1 2 3 4 5 6 7 8 9 10	*l. Você sentiu que foi difícil atender às suas próprias necessidades físicas básicas (banheiro acessível, chuveiros, refeições a preço acessíveis, lugar onde ficar, estacionamento, etc.)?*<br>*Nunca Às vezes Frequentemente Sempre*<br>*1 2 3 4*<br>*Como você AVALIARIA este aspecto da experiência que você e seu filho tiveram na UTI?*<br>*Terrível Quase ideal*<br>*1 2 3 4 5 6 7 8 9 10*
m. Were you able to be physically close to your child as much as you would have liked?<br>Never Sometimes Often Always<br>1 2 3 4<br>How would you RATE this aspect of the experience you and your child had in the ICU?<br>Terrible Almost ideal<br>1 2 3 4 5 6 7 8 9 10	*m. Você foi capaz de ficar fisicamente próximo de seu filho (a) o quanto gostaria de ter ficado?*<br>*Nunca Às vezes Frequentemente Sempre*<br>*1 2 3 4*<br>*Como você AVALIARIA este aspecto da experiência que você e seu filho tiveram na UTI?*<br>*Terrível Quase ideal*<br>*1 2 3 4 5 6 7 8 9 10*
n. Were hospital clergy or chaplains available the way that you wanted them to be?<br>Never Sometimes Often Always<br>1 2 3 4<br>How would you RATE this aspect of the experience you and your child had in the ICU?<br>Terrible Almost ideal<br>1 2 3 4 5 6 7 8 9 10	*n. Havia líderes religiosos disponíveis da maneira que você quisesse que eles estivessem?*<br>*Nunca Às vezes Frequentemente Sempre*<br>*1 2 3 4*<br>*Como você AVALIARIA este aspecto da experiência que você e seu filho tiveram na UTI?*<br>*Terrível Quase ideal*<br>*1 2 3 4 5 6 7 8 9 10*
o. Did clinical staff respect your family's spiritual and/or religious needs?<br>Never Sometimes Often Always<br>1 2 3 4<br>How would you RATE this aspect of the experience you and your child had in the ICU?<br>Terrible Almost ideal<br>1 2 3 4 5 6 7 8 9 10	*o. A equipe clínica respeitou as necessidades espirituais e/ou religiosas de sua família?*<br>*Nunca Às vezes Frequentemente Sempre*<br>*1 2 3 4*<br>*Como você AVALIARIA este aspecto da experiência que você e seu filho tiveram na UTI?*<br>*Terrível Quase ideal*<br>*1 2 3 4 5 6 7 8 9 10*
p. Did you feel that clinical staff prepared you for what might happen to your child?<br>Never Sometimes Often Always<br>1 2 3 4<br>How would you RATE this aspect of the experience you and your child had in the ICU?<br>Terrible Almost ideal<br>1 2 3 4 5 6 7 8 9 10	*p. Você sentiu que a equipe clínica preparou você para o que pudesse acontecer com seu filho (a)?*<br>*Nunca Às vezes Frequentemente Sempre*<br>*1 2 3 4*<br>*Como você AVALIARIA este aspecto da experiência que você e seu filho tiveram na UTI?*<br>*Terrível Quase ideal*<br>*1 2 3 4 5 6 7 8 9 10*
2. Was your child in the ICU for more than 12 hours? (please circle yes or no)<br>Yes No<br>If yes, answers Question a below<br>If No, continue on the next page with Question 3	*2. Seu filho (a) ficou na UTI por mais de 12 horas? (Por favor circule sim ou não)*<br>*Sim Não*<br>*Para Sim, responda a Pergunta "a" abaixo*<br>*Para Não, continue na próxima página na Pergunta 3*
a. Did you feel that nurses and doctors did a good job of passing information about your child on to the next shift or rotation?<br>Never Sometimes Often Always<br>1 2 3 4<br>How would you RATE this aspect of the experience you and your child had in the ICU?<br>Terrible Almost ideal<br>1 2 3 4 5 6 7 8 9 10	*a. Você achou que as enfermeiras e os médicos fizeram um bom trabalho ao passarem informações sobre seu filho (a) para o turno seguinte ou na troca de cuidador?*<br>*Nunca Às vezes Frequentemente Sempre*<br>*1 2 3 4*<br>*Como você AVALIARIA este aspecto da experiência que você e seu filho tiveram na UTI?*<br>*Terrível Quase ideal*<br>*1 2 3 4 5 6 7 8 9 10*

Continue...

...continuation

ORIGINAL	BRAZILIAN VERSION
3. While in the ICU during the last three days of your child's life…	*3. Na UTI durante os últimos três dias de vida de seu filho (a) ...*
a. Were you given enough privacy with your child near the end of your child's life?	*a. Você teve privacidade suficiente com seu filho(a) perto do final de vida dele?*
Yes No Don't know	*Sim Não Não sei*
1 2 0	*1 2 0*
How would you RATE this aspect of the experience you and your child had in the ICU?	*Como você AVALIARIA este aspecto da experiência que você e seu filho tiveram na UTI?*
Terrible Almost ideal	*Terrível Quase ideal*
1 2 3 4 5 6 7 8 9 10	*1 2 3 4 5 6 7 8 9 10*
b. Did clinical staff help you create memories (such as handprints, lockets of hair, Photographs) of your child?	*b. A equipe clínica ajudou você a criar lembranças (tais como impressão das mãos, mechas de cabelo, fotografias) de seu filho(a)?*
Yes No Don't know	*Sim Não Não sei*
1 2 0	*1 2 0*
Terrible Almost ideal	*Terrível Quase ideal*
1 2 3 4 5 6 7 8 9 10	*1 2 3 4 5 6 7 8 9 10*
c. Once your child died, were you allowed to stay with him/her for as long as you wanted?	*c. Quando seu filho (a) morreu, permitiram que você ficasse com ele/ela pelo tempo que você quisesse?*
Yes No Don't know	*Sim Não Não sei*
1 2 0	*1 2 0*
Terrible Almost ideal	*Terrível Quase ideal*
1 2 3 4 5 6 7 8 9 10	*1 2 3 4 5 6 7 8 9 10*
**SECTION B**	***SEÇÃO B***
4. Here are some additional questions about your experiences in the pediatric ICU. Some of these questions may sound very similar to questions we've asked you previously. Please bear with us. Asking questions in different ways will help us to better understand different aspects of your experience.<br>The next group of questions are about how you and your child were cared for by clinical staff while in the ICU, and your satisfaction with that care.	*4. Temos aqui algumas perguntas adicionais sobre suas experiências na UTI pediátrica. Algumas destas perguntas podem parecer muito similares às perguntas que fizemos anteriormente. Por favor tenha paciência conosco. Fazer perguntas em diferentes maneiras nos ajudará a compreender melhor os diferentes aspectos de sua experiência.*<br>*O próximo grupo de perguntas será sobre como nossa equipe clínica cuidou de você e de seu filho (a) enquanto ele (a) estava na UTI, e sobre a satisfação com os cuidados.*
How would you rate…	*Como você avaliaria...*
a. The courtesy, respect and compassion that your child was given while in the ICU?	*a. Cortesia, respeito e compaixão que seu filho (a) recebeu enquanto estava na UTI?*
Excellent Very good Good Fair Poor Doesn't apply	*Excelente Muito bom Bom Razoável Péssimo Não se aplica*
1 2 3 4 5 6	*1 2 3 4 5 6*
b. The courtesy, respect and compassion you were given by the ICU staff?	*b. Cortesia, respeito e compaixão que você recebeu pela equipe da UTI?*
Excellent Very good Good Fair Poor Doesn't apply	*Excelente Muito bom Bom Razoável Péssimo Não se aplica*
1 2 3 4 5 6	*1 2 3 4 5 6*
c. The completeness of information given to you by ICU staff? (for example, information about what was happening to your child and why things were being done)	*c. A integridade das informações dadas a você pela equipe da UTI (por exemplo, informação sobre o que estava acontecendo com seu filho (a) e porque as coisas estavam sendo feitas)*
Excellent Very good Good Fair Poor Doesn't apply	*Excelente Muito bom Bom Razoável Péssimo Não se aplica*
1 2 3 4 5 6	*1 2 3 4 5 6*
5. Some people want everything done for their health problems while others do not want a lot done. How satisfied were you with the level or amount of care your child received in the ICU?<br>Please circle your answer.	*5. Algumas pessoas querem que tudo seja feito pelos problemas de saúde delas, enquanto outras pessoas não querem que muito seja feito. Qual o seu grau de satisfação com o nível ou quantidade de cuidados que seu filho(a) recebeu na UTI?*<br>*Por favor, circule sua resposta*
Very dissatisfied / Slightly dissatisfied / Mostly satisfied / Very satisfied / Completely satisfied	*Muito insatisfeito / Um pouco insatisfeito / Satisfeito em geral / Muito satisfeito / Completamente satisfeito*
6. The next few questions ask about the emotional support provided while your child was in the ICU. Please circle one answer for each question.	*6. As próximas perguntas são sobre o apoio emocional recebido enquanto seu filho (a) estava na UTI. Por favor circule uma resposta para cada pergunta.*
a. While your child was in intensive care, did someone talk with you about your religious or spiritual beliefs?	*a. Enquanto seu filho (a) estava na terapia intensiva, alguém falou com você sobre suas crenças religiosas ou espirituais?*
No Yes	*Não Sim*
If No, skip to question b	*Se Não, pule para pergunta b*
If Yes – Was this done in a sensitive manner?	*Se Sim – Isso foi feito de uma maneira sensível?*
No Yes	*Não Sim*
If no, skip to question b	*Se Não, pule para pergunta b*
If Yes – Did you have as much contact of that kind (talk about religious/spiritual beliefs) as you wanted while your child was in intensive care?	*Se Sim – Você teve bastante contato desse tipo (falar sobre suas crenças religiosas/ espirituais) do modo que gostaria enquanto seu filho estava na terapia intensiva?*
No Yes	*Não Sim*
b. How much support in dealing with your feelings about your child's death did the doctors, nurses, and other professional staff taking care of him/her provide you?	*b. Quanto suporte para lidar com os sentimentos sobre a morte de seu filho (a) os médicos, enfermeiras, e outros profissionais da equipe que cuidaram dele (a) forneceram a você?*
Less than was needed / The right amount / More than was needed	*Menos do que necessário / A quantidade certa / Mais do que necessário*

Continue...

...continuation

ORIGINAL			BRAZILIAN VERSION		
c. Did a doctor, nurse or other professional staff taking care of your child talk about how you might feel after his/her death?			*c. Algum médico, enfermeira ou outro profissional da equipe que cuidava de seu filho (a) falou sobre como você poderia se sentir depois da morte dele (a)?*		
No		Yes	*Não*		*Sim*
If no			*Se não*		
Would you have wanted them to?			*Você gostaria que eles tivessem falado?*		
No		Yes	*Não*		*Sim*
If yes			*Se sim*		
Was it done in a sensitive manner?			*Isso foi feito de maneira sensível?*		
No		Yes	*Não*		*Sim*
d. Did a doctor, nurse, or other professional staff taking care of your child suggest someone you could turn to for help if you were feeling stressed?			*d. Algum médico, enfermeira ou outro profissional da equipe que cuidava de seu filho (a) sugeriu alguém a quem você pudesse pedir ajuda se você se sentisse estressado?*		
No		Yes	*Não*		*Sim*
7. The next few questions ask about receiving information and making decisions while you and your child were in the ICU. Please circle one answer for each question.			*7. As próximas perguntas são sobre receber informações e tomar decisões enquanto você e seu filho (a) estavam na UTI. Por favor circule uma resposta para cada pergunta.*		
a. While your child was in intensive care, did you talk with any of your child's doctors yourself?			*a. Enquanto seu filho (a) estava na terapia intensiva, você mesmo conversou com algum dos médicos do seu filho (a)?*		
No		Yes	*Não*		*Sim*
If no, skip to question b			*Se não, pule para a pergunta b*		
If yes			*Se sim*		
Was there ever a problem understanding what any doctor was saying to you about what to expect form treatment?			*Houve algum problema de compreensão sobre o que algum dos médicos lhe dizia sobre o que esperar do tratamento?*		
No		Yes	*Não*		*Sim*
If yes			*Se sim*		
Did you feel that the doctors you talked to listened to your concerns about your child's medical treatment?			*Você sentiu que os médicos com os quais você falou, ouviram suas preocupações sobre o tratamento médico de seu filho?*		
No		Yes	*Não*		*Sim*
If no – skip to question b			*Se não - pule para a pergunta b*		
Had no concerns – skip to question b			*Não tive preocupações – pule para a questão b*		
If yes			*Se sim*		
How much information did the doctors provide you about your child's medical condition?			*Quanta informação os médicos lhe deram sobre a condição médica de seu filho?*		
Less than was needed	The right amount	More than was needed	*Menos do que necessário*	*A quantidade certa*	*Mais do que necessário*
b. Was there ever a decision made about your child's care without enough input from you, your child or your family?			*b. Alguma decisão foi tomada quanto ao seu filho(a) sem um parecer seu, de seu filho ou de sua família?*		
No		Yes	*Não*		*Sim*
c. Did you or your family receive any information about what to expect while your child was dying?			*c. Você ou sua família recebeu alguma informação sobre o que esperar enquanto seu filho (a) estava morrendo?*		
No		Yes	*Não*		*Sim*
If No			*Se Não*		
Would you have wanted some information about that?			*Você gostaria de ter recebido alguma informação sobre isso?*		
No		Yes	*Não*		*Sim*
If Yes			*Se Sim*		
Would you have wanted more information about that?					
No		Yes	*Não*		*Sim*
Você gostaria de ter recebido mais informações sobre isso?					
No		Yes	*Não*		*Sim*
d. Did you or your Family receive any information about what to do at the time of your child's death?			*d. Você ou sua Família recebeu alguma informação sobre o que fazer no momento da morte de seu filho (a)?*		
No		Yes	*Não*		*Sim*
If No			*Se Não*		
Would you have wanted some information about that?			*Você gostaria de ter recebido alguma informação sobre isso?*		
No		Yes	*Não*		*Sim*
If Yes			*Se Sim*		
Would you have wanted more information about that?			*Você gostaria de ter recebido mais informações sobre isso?*		
No		Yes	*Não*		*Sim*

Continue...

...continuation

ORIGINAL	BRAZILIAN VERSION
e. Did you or your family receive any information about the medicines that would be used to manage your child's pain, shortness of breath, or other symptoms?<br>No Yes<br>If No<br>Would you have wanted some information about that?<br>No Yes<br>If Yes<br>Would you have wanted more information about that?<br>No Yes	*e. Você ou sua família recebeu alguma informação sobre os remédios que seriam usados para controlar a dor, falta de ar, ou outros sintomas de seu filho (a)?*<br>*Não Sim*<br>*Se Não*<br>*Você gostaria de ter recebido alguma informação sobre isso?*<br>*Não Sim*<br>*Se Sim*<br>*Você gostaria de ter recebido mais informações sobre isso?*<br>*Não Sim*
f. How often were you or other Family members kept informed about your child's condition?<br>Always Usually Sometimes Never	*f. Com que frequência você ou outros membros da Família foram informados sobre as condições de seu filho (a)?*<br>*Sempre Geralmente Às vezes Nunca*
8. The next few questions ask about how well-coordinated the care was in the ICU. Please circle one answer for each question.	*8. As próximas perguntas são sobre se você acha que os cuidados da UTI foram bem coordenados. Por favor circule uma resposta para cada pergunta.*
a. How often did any doctor give confusing or contradictory information about your child's medical treatment?<br>Always Usually Sometimes Never	*a. Com que frequência qualquer um dos médicos lhe deu informações confusas ou contraditórias sobre o tratamento médico de seu filho (a)?*<br>*Sempre Geralmente Às vezes Nunca*
b. Was there always a doctor in charge of your child's care?<br>No Yes	*b. Sempre houve um médico responsável pelos cuidados de seu filho (a)?*<br>*Não Sim*
c. Was it always clear to you which doctor was in charge of your child's care?<br>No Yes	*c. Sempre ficou claro para você qual médico era o responsável pelos cuidados de seu filho (a)?*<br>*Não Sim*
d. Did your child receive too much, too little, or just the right amount of medication for your child's pain?<br>Less than was needed The right amount More than was needed	*d. Seu filho (a) recebeu medicação demais, muito pouco, ou a quantidade certa para a dor dele(a)?*<br>*Menos do que necessário A quantidade certa Mais do que necessário*
e. Was there any problem with doctors or nurses not knowing enough about your child's medical history to provide the best possible care?<br>No Yes	*e. Houve algum problema com os médicos ou enfermeiras por não saberem o suficiente sobre a história médica de seu filho (a) para oferecer o melhor cuidado possível?*<br>*Não Sim*
9. We would like to know more about the communication you had with the clinicians who took care of your child during the last 3 days in the ICU. How much of the communication that you had with doctors, nurses, or other clinical staff took place in:	*9. Nós gostaríamos de saber mais sobre a comunicação que você teve com os clínicos que cuidaram de seu filho (a) durante os 3 últimos dias na UTI. Quanto de comunicação que você teve com médicos, enfermeiras, ou outra equipe médica ocorreu em:*
a. Formal family conferences<br>None Less than half About half More than half Most<br>1 2 3 4 5	*a. Reuniões formais com a família*<br>*Nenhuma Menos que a metade Metade Mais do que a metade A maior parte*<br>*1 2 3 4 5*
b. Discussion with you during "rounds"<br>None Less than half About half More than half Most<br>1 2 3 4 5	*b. Discussões com você durante as "visitas médicas"*<br>*Nenhuma Menos que a metade Metade Mais do que a metade A maior parte*<br>*1 2 3 4 5*
c. Informal private conversations with you at the bedside?<br>None Less than half About half More than half Most<br>1 2 3 4 5	*c. Conversa informal particular com você à beira do leito?*<br>*Nenhuma Menos que a metade Metade Mais do que a metade A maior parte*<br>*1 2 3 4 5*
d. Informal discussion with you in non-private places, such as hallways and waiting areas<br>None Less than half About half More than half Most<br>1 2 3 4 5	*d. Discussão informal com você em lugares não privados, tais como corredores e áreas de espera.*<br>*Nenhuma Menos que a metade Metade Mais do que a metade A maior parte*<br>*1 2 3 4 5*
e. Telephone calls<br>None Less than half About half More than half Most<br>1 2 3 4 5	*e. Ligações telefônicas*<br>*Nenhuma Menos que a metade Metade Mais do que a metade A maior parte*<br>*1 2 3 4 5*
f. Other (Please specify ____________________)<br>None Less than half About half More than half Most<br>1 2 3 4 5	*f. Outro (Por favor especifique ____________________)*<br>*Nenhuma Menos que a metade Metade Mais do que a metade A maior parte*<br>*1 2 3 4 5*

Continue...

...continuation

ORIGINAL	BRAZILIAN VERSION
10. Now please think about all of the communication you had during your child's last 3 days with clinicians caring for your child. How would you rate each type of communication?	*10. Agora por favor pense em toda a comunicação que você teve durante os últimos 3 dias de seu filho (a) com os clínicos que cuidavam dele. Como você avaliaria cada tipo de comunicação?*
a. Formal family conferences<br>None 1 / Less than half 2 / About half 3 / More than half 4 / Most 5	*a. Reuniões formais com a família*<br>*Nenhuma 1 / Menos que a metade 2 / Metade 3 / Mais do que a metade 4 / A maior parte 5*
b. Discussion with you during "rounds"<br>None 1 / Less than half 2 / About half 3 / More than half 4 / Most 5	*b. Discussões com você durante as "visitas médicas"*<br>*Nenhuma 1 / Menos que a metade 2 / Metade 3 / Mais do que a metade 4 / A maior parte 5*
c. Informal private conversations with you at the bedside?<br>None 1 / Less than half 2 / About half 3 / More than half 4 / Most 5	*c. Conversa informal particular com você à beira do leito?*<br>*Nenhuma 1 / Menos que a metade 2 / Metade 3 / Mais do que a metade 4 / A maior parte 5*
d. Informal discussion with you in non-private places, such as hallways and waiting areas<br>None 1 / Less than half 2 / About half 3 / More than half 4 / Most 5	*d. Discussão informal com você em lugares não privados, tais como corredores e áreas de espera*<br>*Nenhuma 1 / Menos que a metade 2 / Metade 3 / Mais do que a metade 4 / A maior parte 5*
e. Telephone calls<br>None 1 / Less than half 2 / About half 3 / More than half 4 / Most 5	*e. Ligações telefônicas*<br>*Nenhuma 1 / Menos que a metade 2 / Metade 3 / Mais do que a metade 4 / A maior parte 5*
f. Other (Please specify__________________)<br>None 1 / Less than half 2 / About half 3 / More than half 4 / Most 5	*f. Outro (Por favor especifique __________________)*<br>*Nenhuma 1 / Menos que a metade 2 / Metade 3 / Mais do que a metade 4 / A maior parte 5*
For the next set of questions, please circle your answer:	*Para a próxima sequência de perguntas, por favor circule sua resposta:*
11. How many family conference did you participate in during the last 3 days of your child's time in the ICU?<br>0 / 1 / 2 - 3 / 4 or more	*11. Quantas reuniões de família você participou durante os 3 últimos dias de permanência de seu filho (a) na UTI?*<br>*0 / 1 / 2 - 3 / 4 ou mais*
12. Were any staff other than doctors involved in any of the family conferences that you attended?<br>Yes / No / Not sure	*12. Alguma outra equipe que não médicos se envolveu em alguma reunião de família na qual você participou?*<br>*Sim / Não / Não tenho certeza*
13. Were you present during a CPR procedure (a resuscitation attempt) that was performed on your child during his/her time in the ICU?<br>Yes / No / Not sure	*13. Você estava presente durante um procedimento de RCP (tentativa de ressuscitação) administrado em seu filho (a) durante a permanência dele na UTI?*<br>*Sim / Não / Não tenho certeza*
14. Were you present during invasive procedure (for example, insertion of a chest tube or breathing tube) that was performed on your child during his/her time in the ICU?<br>Yes / No / Not sure	*14. Você estava presente durante um procedimento invasivo (por exemplo, inserção de um tubo no tórax ou tubo de respiração) que foi feito em seu filho (a) durante a permanência dele na UTI?*<br>*Sim / Não / Não tenho certeza*
15. How willing were staff to work around or "bend the rules" to meet the needs of your child and those of your family?<br>Very willing / Somewhat willing / Unwilling	*15. Quão disposta foi a equipe para se esforçar ou "dar um jeito" para atender as necessidades de seu filho (a) e as da sua família?*<br>*Muito disposta / Um pouco disposta / Nada disposta*
16. While your child was in the ICU did anyone talk to you about grief and bereavement support that might be available to your family?<br>Yes / No / Not sure	*16. Enquanto seu filho (a) esteve na UTI, alguém falou com você sobre apoio à perda e o suporte ao luto que poderia estar disponível à sua família?*<br>*Sim / Não / Não tenho certeza*
17. While your child in the ICU, were given any written materials on grief bereavement?<br>Yes / No / Not sure	*17. Enquanto seu filho esteve na UTI, vocês receberam algum material escrito sobre a dor da perda e luto?*<br>*Sim / Não / Não tenho certeza*
18. What types of contacts have you had with the ICU sttaf since your child died? (Please check all that apply)<br>_____ staff sent a card<br>_____ staff called on the phone<br>_____ staff attended your child's funeral<br>_____ staff mailed out information about bereavement support<br>_____ I returned to the hospital to meet with the ICU staff<br>_____ I returned to the hospital to visit ICU staff<br>_____ Other	*18. Que tipo de contato você teve com a equipe da UTI desde que seu filho morreu? (Por favor marque todos que se aplicam)*<br>*_____ a equipe enviou um cartão*<br>*_____ a equipe fez uma ligação telefônica*<br>*_____ a equipe esteve no funeral de seu filho*<br>*_____ a equipe enviou informação sobre o suporte ao luto pelo correio*<br>*_____ eu voltei ao hospital para me encontrar com a equipe da UTI*<br>*_____ eu voltei ao hospital para visitar a equipe da UTI*<br>*_____ Outro*

Continue...

...continuation

ORIGINAL	BRAZILIAN VERSION
**SECTION C**	***SEÇÃO C***
Now we want ask you some questions that you can answer in your own words.	*Agora, queremos fazer algumas perguntas que você pode responder com suas próprias palavras.*
19. While your child was in intensive care, what do you wish could have been done differently, and why?	*19. Enquanto seu filho (a) esteve na unidade de terapia intensiva, o que você gostaria que pudesse ter sido feito de diferente, e por quê?*
20. What changes in the care provided to children in the intensive care at your hospital would you recommend, and why?	*20. Que mudanças no cuidado dado às crianças na terapia intensiva em seu hospital você recomendaria, e por quê?*
This completes the survey.<br>Please return it in the envelop provided.<br>THANK YOU VERY MUCH FOR YOUR HELP.	*Aqui termina a pesquisa.*<br>*Por favor, retorne-a no envelope fornecido.*<br>*MUITO OBRIGADO POR SUA AJUDA.*

Table 2S - Original instrument PICU-QODD and Brazilian version - Multidisciplinary Team

ORIGINAL	BRAZILIAN VERSION
PICU-QODD: A Child's and his/her parent's experience at the end of life in pediatric intensive care. A survey of clinicians	*PICU-QODD: Experiência de uma criança e de seus pais no fim de vida em terapia intensiva pediátrica. Uma pesquisa com os clínicos*
You recently cared for a child whom died in the intensive care unit (ICU). This survey is about the experiences of that child and his/her parents in intensive care near the end of the child's life. (The gender, age, and date of death of this patient are listed in the attached cover letter.) You cared for this patient during their final hours and/or days of life, so we are interested in your assessment of these experience. Your input, along with that of your colleagues, will help to describe and ultimately improve the end-of-life care provided for patients and their families in the pediatric intensive care setting. We expect that it will take 10 – 15 minutes to complete the survey.<br>We appreciate your honesty in your responses. Please be assured that the answer you provide will be Kept entirely confidential.	*Você recentemente cuidou de uma criança que morreu na unidade de terapia intensiva (UTI). Esta pesquisa é sobre as experiências dessa criança e de seus pais em terapia intensiva perto do final de vida da criança. (O sexo, idade e data da morte deste paciente estão listados na carta anexa). Você cuidou desse paciente durante suas últimas horas e/ou dias de vida, por isso estamos interessados na sua avaliação dessas experiências. Suas informações, juntamente com as de seus colegas, ajudarão a descrever e a melhorar os cuidados de fim de vida prestados aos pacientes e suas famílias no contexto de cuidados intensivos pediátricos. Estimamos que esta pesquisa leve de 10 a 15 minutos para ser concluída.*<br>*Agradecemos sua honestidade nas respostas. Tenha certeza de que a resposta que você fornecer será mantida totalmente confidencial.*
THANK YOU FOR YOUR HELP	*OBRIGADA POR SUA AJUDA*
**SECTION A**	***SEÇÃO A***
1. You recently cared for a child who died in the ICU. The age, gender, and date of death of the child are noted on the attached cover letter.<br>The following questions are about experiences this child and his/her parents may have had in the ICU during the child's last 3 days of life. Please think about the last 3 days of the child's life, even if the ICU stay was longer than 3 days. If the child was in the ICU less than 3 days, consider only the time in the ICU. "Parents" refers to parents or any family member or individual serving in that role. "Staff" refers to any clinical staff, e.g., nurses, doctors, respiratory therapists, chaplains, social workers, child life specialists, psychologists, etc., who cared for the child and/or the family. In answering these questions, please base your ratings on how you think these experiences affected the quality of dying and death for the child and his/her parents, not how you think the parents (or the child if able) would have rated these experiences. We understand that you were not present the whole time, but please make your best estimate. On the rating scale below, 0 = "a terrible experience" and 10 = "an almost perfect experience". If the child or his/her parents did not have, or did not appear to have, a particular experience or if you do not know enough to rate it, you may check the box on the right.	*1. Você recentemente cuidou de uma criança que morreu na UTI. A idade, sexo e data da morte da criança estão anotados na carta de apresentação anexa. As perguntas a seguir são sobre experiências que esta criança e seus pais podem ter tido na UTI durante os últimos 3 dias de vida da criança. Por favor pense nos últimos 3 dias da vida da criança, mesmo que a permanência na UTI tenha durado mais de 3 dias. Se a criança esteve na UTI menos de 3 dias, considere apenas o tempo na UTI. A palavra "Pais" refere-se aos pais ou a qualquer membro da família ou indivíduo que exerça esse papel. "Equipe" refere-se a qualquer grupo clínico, por exemplo enfermeiros, médicos, fisioterapeutas, sacerdotes, assistentes sociais, especialistas em pediatria, psicólogos, etc., que cuidaram da criança e/ou da família. Ao responder estas perguntas, por favor baseie suas avaliações em como você acha que essas experiências afetaram a qualidade de morrer e de morte para a criança e seus pais, e não como você acha que os pais (ou a criança, se capaz) teriam avaliado essas experiências. Entendemos que você não esteve presente o tempo todo, mas faça sua melhor estimativa. Na escala de classificação abaixo, 0 = "experiência terrível" e 10 = "experiência quase perfeita". Se a criança ou seus pais não tiveram, ou não pareceram ter, uma experiência em particular ou se você não sabe o suficiente para avaliá-la, você pode marcar no espaço à direita.*
a. The child was free of pain<br>Terrible experience — Almost perfect experience — Don't know<br>0 1 2 3 4 5 6 7 8 9 10 ☐	*a. A criança estava livre de dor*<br>*Experiência terrível — Experiência quase perfeita — Não sei*<br>*0 1 2 3 4 5 6 7 8 9 10* ☐
b. The child was free of other troubling symptoms<br>Terrible experience — Almost perfect experience — Don't know<br>0 1 2 3 4 5 6 7 8 9 10 ☐	*b. A criança estava livre de outros sintomas preocupantes*<br>*Experiência terrível — Experiência quase perfeita — Não sei*<br>*0 1 2 3 4 5 6 7 8 9 10* ☐
c. Staff responded quickly to parents' concerns about their child's symptoms<br>Terrible experience — Almost perfect experience — Don't know<br>0 1 2 3 4 5 6 7 8 9 10 ☐	*c. A equipe respondeu rapidamente às preocupações dos pais quanto aos sintomas da criança*<br>*Experiência terrível — Experiência quase perfeita — Não sei*<br>*0 1 2 3 4 5 6 7 8 9 10* ☐

Continue...

...continuation

ORIGINAL	BRAZILIAN VERSION
d. Staff gave parents information about their child in a way that they could understand<br>Terrible experience — Almost perfect experience — Don't know<br>0 1 2 3 4 5 6 7 8 9 10 ☐	*d. A equipe deu informações aos pais sobre seu filho de uma maneira que eles pudessem entender*<br>*Experiência terrível — Experiência quase perfeita — Não sei*<br>*0 1 2 3 4 5 6 7 8 9 10* ☐
e. Staff created an atmosphere in which parents felt comfortable asking questions about their child<br>Terrible experience — Almost perfect experience — Don't know<br>0 1 2 3 4 5 6 7 8 9 10 ☐	*e. A equipe criou um ambiente em que os pais se sentissem confortáveis fazendo perguntas sobre seu filho*<br>*Experiência terrível — Experiência quase perfeita — Não sei*<br>*0 1 2 3 4 5 6 7 8 9 10* ☐
f. Staff demonstrated that they cared about the child as an individual<br>Terrible experience — Almost perfect experience — Don't know<br>0 1 2 3 4 5 6 7 8 9 10 ☐	*f. A equipe demonstrou que se preocupava com a criança como um ser humano*<br>*Experiência terrível — Experiência quase perfeita — Não sei*<br>*0 1 2 3 4 5 6 7 8 9 10* ☐
g. Staff supported the parents emotionally<br>Terrible experience — Almost perfect experience — Don't know<br>0 1 2 3 4 5 6 7 8 9 10 ☐	*g. A equipe apoiou os pais emocionalmente*<br>*Experiência terrível — Experiência quase perfeita — Não sei*<br>*0 1 2 3 4 5 6 7 8 9 10* ☐
h. Staff discovered and respected parents' wishes and decisions<br>Terrible experience — Almost perfect experience — Don't know<br>0 1 2 3 4 5 6 7 8 9 10 ☐	*h. A equipe descobriu e respeitou os desejos e decisões dos pais*<br>*Experiência terrível — Experiência quase perfeita — Não sei*<br>*0 1 2 3 4 5 6 7 8 9 10* ☐
i. Staff offered parents opportunities to discuss options about their child's care with the healthcare team<br>Terrible experience — Almost perfect experience — Don't know<br>0 1 2 3 4 5 6 7 8 9 10 ☐	*i. A equipe ofereceu aos pais oportunidades para discutir opções sobre o cuidado de seu filho com a equipe de saúde*<br>*Experiência terrível — Experiência quase perfeita — Não sei*<br>*0 1 2 3 4 5 6 7 8 9 10* ☐
j. There were no conflicts between staff and parents about the best way to care for the child<br>Terrible experience — Almost perfect experience — Don't know<br>0 1 2 3 4 5 6 7 8 9 10 ☐	*j. Não houve conflitos entre a equipe e os pais sobre a melhor maneira de cuidar da criança*<br>*Experiência terrível — Experiência quase perfeita — Não sei*<br>*0 1 2 3 4 5 6 7 8 9 10* ☐
k. Parents found it easy to meet their basic physical needs (accessible bathroom, showers, affordable meals, places to stay, parking, etc.)<br>Terrible experience — Almost perfect experience — Don't know<br>0 1 2 3 4 5 6 7 8 9 10 ☐	*k. Os pais acharam fácil atender às suas necessidades físicas básicas (banheiro acessível, chuveiros, refeições a preço acessíveis, lugar onde ficar, estacionamento, etc.)*<br>*Experiência terrível — Experiência quase perfeita — Não sei*<br>*0 1 2 3 4 5 6 7 8 9 10* ☐
l. Staff provided parents with opportunities to be near their child<br>Terrible experience — Almost perfect experience — Don't know<br>0 1 2 3 4 5 6 7 8 9 10 ☐	*l. A equipe deu oportunidade aos pais para ficarem perto da criança*<br>*Experiência terrível — Experiência quase perfeita — Não sei*<br>*0 1 2 3 4 5 6 7 8 9 10* ☐
m. Hospital clergy or chaplains were available<br>Terrible experience — Almost perfect experience — Don't know<br>0 1 2 3 4 5 6 7 8 9 10 ☐	*m. Líderes religiosos do hospital estavam disponíveis*<br>*Experiência terrível — Experiência quase perfeita — Não sei*<br>*0 1 2 3 4 5 6 7 8 9 10* ☐
n. Staff discovered and respected the family's spiritual and/or religious needs<br>Terrible experience — Almost perfect experience — Don't know<br>0 1 2 3 4 5 6 7 8 9 10 ☐	*n. A equipe descobriu e respeitou as necessidades espirituais e/ou religiosas da família*<br>*Experiência terrível — Experiência quase perfeita — Não sei*<br>*0 1 2 3 4 5 6 7 8 9 10* ☐
o. Staff helped parents find ways to touch, hold, and/or connect with their child<br>Terrible experience — Almost perfect experience — Don't know<br>0 1 2 3 4 5 6 7 8 9 10 ☐	*o. A equipe ajudou os pais a encontrarem maneiras de tocar, segurar e/ou conectar-se com o filho (a)*<br>*Experiência terrível — Experiência quase perfeita — Não sei*<br>*0 1 2 3 4 5 6 7 8 9 10* ☐

Continue...

...continuation

ORIGINAL	BRAZILIAN VERSION
p. Staff prepared parents for what might happen to their child<br>Terrible experience Almost perfect experience Don't know<br>0 1 2 3 4 5 6 7 8 9 10 ☐	*p. A equipe preparou os pais quanto ao que poderia acontecer com o filho (a) deles (a)*<br>*Experiência terrível Experiência quase perfeita Não sei*<br>*0 1 2 3 4 5 6 7 8 9 10* ☐
q. Staff provided parents and their child with privacy near the end of their child's life<br>Terrible experience Almost perfect experience Don't know<br>0 1 2 3 4 5 6 7 8 9 10 ☐	*q. A equipe forneceu privacidade aos pais e seu filho (a) no fim de vida*<br>*Experiência terrível Experiência quase perfeita Não sei*<br>*0 1 2 3 4 5 6 7 8 9 10* ☐
r. Staff helped parents create memories (such as handprints, lockets of hair, photographs) of their child<br>Terrible experience Almost perfect experience Don't know<br>0 1 2 3 4 5 6 7 8 9 10 ☐	*r. A equipe ajudou os pais a criarem lembranças (tais como impressão das mãos, mechas de cabelo, fotografias) de seu filho (a)*<br>*Experiência terrível Experiência quase perfeita Não sei*<br>*0 1 2 3 4 5 6 7 8 9 10* ☐
s. Once the child died, staff allowed parents to stay with their child as long as they wanted<br>Terrible experience Almost perfect experience Don't know<br>0 1 2 3 4 5 6 7 8 9 10 ☐	*s. Quando a criança morreu, a equipe permitiu que os pais ficassem com ela o tempo que quisessem*<br>*Experiência terrível Experiência quase perfeita Não sei*<br>*0 1 2 3 4 5 6 7 8 9 10* ☐
t. Nurses and doctors did a good job of passing information about the child onto the next shift or rotation (if in the ICU less than 12 hours check Not Applicable<br>Terrible experience Almost perfect experience Don't know<br>0 1 2 3 4 5 6 7 8 9 10 ☐	*t. As enfermeiras e os médicos fizeram um bom trabalho ao passarem informações sobre a criança para o turno seguinte ou na troca de cuidador*<br>*Experiência terrível Experiência quase perfeita Não sei*<br>*0 1 2 3 4 5 6 7 8 9 10* ☐
**SECTION B**	***SEÇÃO B***
2. Still thinking about the last 3 days of the child's life, how would you rate the quality of care given to this child and his/her family in intensive care?<br>Poor Excellent Don't know<br>0 1 2 3 4 5 6 7 8 9 10 ☐	*2. Ainda pensando nos 3 últimos dias de vida da criança, como você avaliaria a qualidade dos cuidados dados a ela e à sua família na terapia intensiva?*<br>*Péssimo Excelente Não sei*<br>*0 1 2 3 4 5 6 7 8 9 10* ☐
3. Overall, how would you rate the quality of the moment of death of this child?<br>Poor Excellent Don't know<br>0 1 2 3 4 5 6 7 8 9 10 ☐	*3. Em geral, como você avaliaria a qualidade do momento de morte dessa criança?*<br>*Péssimo Excelente Não sei*<br>*0 1 2 3 4 5 6 7 8 9 10* ☐
4. These questions focus on specific ways that you or other clinical staff may have helped this patient's family. Not every staff member will have done many or even some of these. For each question, check the box for every response option that applies. You may check more than one response for each question.<br>During the time this child was in the ICU, did you:	*4. Estas perguntas se concentram em formas específicas pelas quais você ou outra equipe clínica pode ter ajudado a família desse paciente. Nem todo membro da equipe terá feito muitas ou mesmo algumas delas. Para cada pergunta, marque a melhor opção de resposta. Você pode marcar mais de uma resposta para cada pergunta.*<br>*Durante o tempo que esta criança esteve na UTI, você:*
a. Explain to the family about the patient's medical equipment and therapies?<br>__ Yes, I did this<br>__ Yes, another clinical staff member did this<br>__ No, not done<br>__ Don't know<br>__ Does not apply for this family<br>__ This is outside the scope of my role	*a. Explicou à família sobre as terapias e os equipamentos médicos do paciente?*<br>__ *Sim, eu fiz isso*<br>__ *Sim, outro membro da equipe clínica fez isso*<br>__ *Não, não foi feito*<br>__ *Não sei*<br>__ *Não se aplica a esta família*<br>__ *Isso não faz parte da minha função*
b. Tell the family what to expect during conferences with the health care team members?<br>__ Yes, I did this<br>__ Yes, another clinical staff member did this<br>__ No, not done<br>__ Don't know<br>__ Does not apply for this family<br>__ This is outside the scope of my role	*b. Disse à família o que esperar durante as reuniões com os membros da equipe de cuidados de saúde?*<br>__ *Sim, eu fiz isso*<br>__ *Sim, outro membro da equipe clínica fez isso*<br>__ *Não, não foi feito*<br>__ *Não sei*<br>__ *Não se aplica a esta família*<br>__ *Isso não faz parte da minha função*

Continue...

...continuation

ORIGINAL	BRAZILIAN VERSION
c. Talk with the family about what the patient valued in life?	*c. Conversou com a família sobre o que o paciente valorizava em vida?*
__ Yes, I did this	*__ Sim, eu fiz isso*
__ Yes, another clinical staff member did this	*__ Sim, outro membro da equipe clínica fez isso*
__ No, not done	*__ Não, não foi feito*
__ Don't know	*__ Não sei*
__ Does not apply for this family	*__ Não se aplica a esta família*
__ This is outside the scope of my role	*__ Isso não faz parte da minha função*
d. Talk with the family about the patient's illness and treatment?	*d. Conversou com a família sobre a doença e o tratamento do paciente?*
__ Yes, I did this	*__ Sim, eu fiz isso*
__ Yes, another clinical staff member did this	*__ Sim, outro membro da equipe clínica fez isso*
__ No, not done	*__ Não, não foi feito*
__ Don't know	*__ Não sei*
__ Does not apply for this family	*__ Não se aplica a esta família*
__ This is outside the scope of my role	*__ Isso não faz parte da minha função*
e. Talk with the family about their feelings?	*e. Conversou com a família sobre os sentimentos deles?*
__ Yes, I did this	*__ Sim, eu fiz isso*
__ Yes, another clinical staff member did this	*__ Sim, outro membro da equipe clínica fez isso*
__ No, not done	*__ Não, não foi feito*
__ Don't know	*__ Não sei*
__ Does not apply for this family	*__ Não se aplica a esta família*
__ This is outside the scope of my role	*__ Isso não faz parte da minha função*
f. Reminisce with the family about the patient?	*f. Relembrou com a família sobre o paciente?*
__ Yes, I did this	*__ Sim, eu fiz isso*
__ Yes, another clinical staff member did this	*__ Sim, outro membro da equipe clínica fez isso*
__ No, not done	*__ Não, não foi feito*
__ Don't know	*__ Não sei*
__ Does not apply for this family	*__ Não se aplica a esta família*
__ This is outside the scope of my role	*__ Isso não faz parte da minha função*
g. Talk with the family about it being all right to talk to and touch their loved one?	*g. Conversou com a família sobre ser normal falar com seu ente querido e tocá-lo?*
__ Yes, I did this	*__ Sim, eu fiz isso*
__ Yes, another clinical staff member did this	*__ Sim, outro membro da equipe clínica fez isso*
__ No, not done	*__ Não, não foi feito*
__ Don't know	*__ Não sei*
__ Does not apply for this family	*__ Não se aplica a esta família*
__ This is outside the scope of my role	*__ Isso não faz parte da minha função*
h. Discuss with the family what the patient might have wanted if he/she were able to participate in the treatment decision making process?	*h. Discutiu com a família sobre o que o paciente desejaria se ele (a) pudesse participar do processo decisório sobre o tratamento?*
__ Yes, I did this	*__ Sim, eu fiz isso*
__ Yes, another clinical staff member did this	*__ Sim, outro membro da equipe clínica fez isso*
__ No, not done	*__ Não, não foi feito*
__ Don't know	*__ Não sei*
__ Does not apply for this family	*__ Não se aplica a esta família*
__ This is outside the scope of my role	*__ Isso não faz parte da minha função*
i. Locate a private place or room for the family to talk among themselves?	*i. Providenciou um lugar ou sala privada para a família conversar entre eles?*
__ Yes, I did this	*__ Sim, eu fiz isso*
__ Yes, another clinical staff member did this	*__ Sim, outro membro da equipe clínica fez isso*
__ No, not done	*__ Não, não foi feito*
__ Don't know	*__ Não sei*
__ Does not apply for this family	*__ Não se aplica a esta família*
__ This is outside the scope of my role	*__ Isso não faz parte da minha função*
j. Talk with the family about any disagreement among the family concerning the plan of care?	*j. Conversou com a família sobre algum desentendimento entre a família com relação ao plano de cuidados?*
__ Yes, I did this	*__ Sim, eu fiz isso*
__ Yes, another clinical staff member did this	*__ Sim, outro membro da equipe clínica fez isso*
__ No, not done	*__ Não, não foi feito*

Continue...

...continuation

ORIGINAL	BRAZILIAN VERSION
__ Don't know<br>__ Does not apply for this family<br>__ This is outside the scope of my role	__ *Não sei*<br>__ *Não se aplica a esta família*<br>__ *Isso não faz parte da minha função*
k. Talk with the family about changes in the patient's plan of care?<br>__ Yes, I did this<br>__ Yes, another clinical staff member did this<br>__ No, not done<br>__ Don't know<br>__ Does not apply for this family<br>__ This is outside the scope of my role	*k. Conversou com a família sobre as mudanças no plano de cuidados do paciente?*<br>__ *Sim, eu fiz isso*<br>__ *Sim, outro membro da equipe clínica fez isso*<br>__ *Não, não foi feito*<br>__ *Não sei*<br>__ *Não se aplica a esta família*<br>__ *Isso não faz parte da minha função*
l. Support the decision/s the family made concerning the patient's care?<br>__ Yes, I did this<br>__ Yes, another clinical staff member did this<br>__ No, not done<br>__ Don't know<br>__ Does not apply for this family<br>__ This is outside the scope of my role	*l. Apoiou a (s) decisão (ões) que a família fez em relação aos cuidados do paciente?*<br>__ *Sim, eu fiz isso*<br>__ *Sim, outro membro da equipe clínica fez isso*<br>__ *Não, não foi feito*<br>__ *Não sei*<br>__ *Não se aplica a esta família*<br>__ *Isso não faz parte da minha função*
m. Talk with the family about their spiritual or religious needs?<br>__ Yes, I did this<br>__ Yes, another clinical staff member did this<br>__ No, not done<br>__ Don't know<br>__ Does not apply for this family<br>__ This is outside the scope of my role	*m. Conversou com a família sobre necessidades espirituais ou religiosas deles?*<br>__ *Sim, eu fiz isso*<br>__ *Sim, outro membro da equipe clínica fez isso*<br>__ *Não, não foi feito*<br>__ *Não sei*<br>__ *Não se aplica a esta família*<br>__ *Isso não faz parte da minha função*
n. Take actions to address the spiritual or religious needs of the family?<br>__ Yes, I did this<br>__ Yes, another clinical staff member did this<br>__ No, not done<br>__ Don't know<br>__ Does not apply for this family<br>__ This is outside the scope of my role	*n. Tomou medidas para atender as necessidades espirituais ou religiosas da família?*<br>__ *Sim, eu fiz isso*<br>__ *Sim, outro membro da equipe clínica fez isso*<br>__ *Não, não foi feito*<br>__ *Não sei*<br>__ *Não se aplica a esta família*<br>__ *Isso não faz parte da minha função*
o. Talk with the family about specific cultural needs?<br>__ Yes, I did this<br>__ Yes, another clinical staff member did this<br>__ No, not done<br>__ Don't know<br>__ Does not apply for this family<br>__ This is outside the scope of my role	*o. Conversou com a família sobre necessidades culturais específicas?*<br>__ *Sim, eu fiz isso*<br>__ *Sim, outro membro da equipe clínica fez isso*<br>__ *Não, não foi feito*<br>__ *Não sei*<br>__ *Não se aplica a esta família*<br>__ *Isso não faz parte da minha função*
p. Take actions to address the cultural needs of the family?<br>__ Yes, I did this<br>__ Yes, another clinical staff member did this<br>__ No, not done<br>__ Don't know<br>__ Does not apply for this family<br>__ This is outside the scope of my role	*p. Tomou medidas para atender as necessidades culturais da família?*<br>__ *Sim, eu fiz isso*<br>__ *Sim, outro membro da equipe clínica fez isso*<br>__ *Não, não foi feito*<br>__ *Não sei*<br>__ *Não se aplica a esta família*<br>__ *Isso não faz parte da minha função*
q. Assure the family that the patient would be kept comfortable?<br>__Yes, I did this<br>__ Yes, another clinical staff member did this<br>__ No, not done<br>__ Don't know<br>__ Does not apply for this family<br>__ This is outside the scope of my role	*q. Assegurou à família de que o paciente seria mantido confortável?*<br>__ *Sim, eu fiz isso*<br>__ *Sim, outro membro da equipe clínica fez isso*<br>__ *Não, não foi feito*<br>__ *Não sei*<br>__ *Não se aplica a esta família*<br>__ *Isso não faz parte da minha função*

Continue...

...continuation

ORIGINAL	BRAZILIAN VERSION
r. Offer additional support to the family?	*r. Ofereceu apoio adicional à família?*
__Yes, I did this	*__ Sim, eu fiz isso*
__ Yes, another clinical staff member did this	*__ Sim, outro membro da equipe clínica fez isso*
__ No, not done	*__ Não, não foi feito*
__ Don't know	*__ Não sei*
__ Does not apply for this family	*__ Não se aplica a esta família*
__ This is outside the scope of my role	*__ Isso não faz parte da minha função*
If yes, please specify________________________________	*Se sim, por favor, especifique__________________________________*
5. Please think about all the communication that you participated in, observed, or were aware of between clinical staff and the family during the child's last 3 days of life. Good communication involves a number of different skills. Overall, how well do you think staff did each of the following?	*5. Por favor pense sobre toda a comunicação da qual você participou, observou, ou esteve ciente entre a equipe clínica e a família durante os 3 últimos dias de vida da criança. Uma boa comunicação envolve várias habilidades diferentes. De modo geral, como você acha que a equipe foi nos itens a seguir?*
a. Built a relationship with the family by, for example, using words that show care and concern, and using tone, pace, eye contact, and posture that show care and concern	*a. Construiu um relacionamento com a família, por exemplo, usando palavras que demonstram cuidado e preocupação, e usando tom, ritmo, contato visual e postura que demonstram cuidado e preocupação*
Poor Fair Good Very good Excellent	*Péssima Razoável Boa Muito boa Excelente*
1 2 3 4 5	*1 2 3 4 5*
b. Gathered information by, for example, using openended questions, clarifying details with more specific or yes/no questions	*b. Coletou informações, por exemplo, usando perguntas abertas, esclarecendo detalhes com perguntas mais específicas ou perguntas de sim/não*
Poor Fair Good Very good Excellent	*Péssima Razoável Boa Muito boa Excelente*
1 2 3 4 5	*1 2 3 4 5*
c. Understood the child's and family's perspective by, for example, asking about life events, circumstances, other people that might affect health, and eliciting patient's and family's beliefs, concerns, and expectations about illness and treatment	*c. Entendeu a perspectiva da criança e da família, por exemplo, perguntando sobre eventos da vida, circunstâncias, outras pessoas que podem afetar a saúde e induzindo crenças, preocupações e expectativas do paciente e da família sobre doença e tratamento*
Poor Fair Good Very good Excellent	*Péssima Razoável Boa Muito boa Excelente*
1 2 3 4 5	*1 2 3 4 5*
d. Shared information by, for example, assessing the child's and family's understanding of problems and desire for more information, explaining using words that family can understand, and asking if the family has questions	*d. Compartilhou informações, por exemplo, avaliando a compreensão da criança e da família sobre os problemas e o desejo de obter mais informações, explicando usando palavras que a família pode entender, e perguntando se a família tem perguntas*
Poor Fair Good Very good Excellent	*Péssima Razoável Boa Muito boa Excelente*
1 2 3 4 5	*1 2 3 4 5*
e. Reached agreement by, for example, including family in choices and decisions to the extent they desire, checking for mutual understanding of diagnostic and/or treatment plans, asking about acceptability of diagnostic and/or treatment plans	*e. Chegou a um acordo, por exemplo, incluindo a família em escolhas e decisões até onde eles desejassem, verificando a compreensão mútua dos planos de diagnóstico e/ou tratamento, perguntando sobre a aceitabilidade dos planos de diagnóstico e/ou tratamento*
Poor Fair Good Very good Excellent	*Péssima Razoável Boa Muito boa Excelente*
1 2 3 4 5	*1 2 3 4 5*
f. Demonstrated empathy by, for example, showing compassion and concern, identifying/labeling/validating the family's emotional responses, and responding appropriately to the child and family's emotional cues	*f. Demonstrou empatia, por exemplo, mostrando compaixão e preocupação, identificando/nomeando/validando as respostas emocionais da família, e respondendo adequadamente às necessidades emocionais da criança e da família*
Poor Fair Good Very good Excellent	*Péssima Razoável Boa Muito boa Excelente*
1 2 3 4 5	*1 2 3 4 5*
g. Communicated accurate information by, for example, accurately conveying the relative seriousness of the child's condition, clearly conveying the expected disease course and explaining options for future care, and giving enough clear information to empower decision making	*g. Comunicou informações precisas, por exemplo, transmitindo com precisão a gravidade relacionada à condição da criança, transmitindo claramente o curso esperado da doença e explicando as opções para futuros cuidados, e fornecendo informações claras o suficiente para fortalecer a tomada de decisões*
Poor Fair Good Very good Excellent	*Péssima Razoável Boa Muito boa Excelente*
1 2 3 4 5	*1 2 3 4 5*
6. We would like to know where communication with the family took place. Thinking about the communication between staff and family in which you participated or observed, how much of that communication took place in:	*6. Gostaríamos de saber onde ocorreu a comunicação com a família. Pensando na comunicação entre a equipe e a família da qual você participou ou observou, o quanto dessa comunicação ocorreu em:*

Continue...

...continuation

ORIGINAL					BRAZILIAN VERSION				
a. Family conferences					*a. Reuniões de família*				
None	Less than half	About half	More than half	Most	*Nenhuma*	*Menos que a metade*	*Metade*	*Mais que a metade*	*A maioria*
1	2	3	4	5	*1*	*2*	*3*	*4*	*5*
b. Discussion with the family during rounds					*b. Discussões com a família durante as visitas médicas*				
None	Less than half	About half	More than half	Most	*Nenhuma*	*Menos que a metade*	*Metade*	*Mais que a metade*	*A maioria*
1	2	3	4	5	*1*	*2*	*3*	*4*	*5*
c. Private conversations with the family at the bedside					*c. Conversas particulares com a família à beira do leito*				
None	Less than half	About half	More than half	Most	*Nenhuma*	*Menos que a metade*	*Metade*	*Mais que a metade*	*A maioria*
1	2	3	4	5	*1*	*2*	*3*	*4*	*5*
d. Non-private places, such as hallways and waiting areas					*d. Lugares não privados, tais como corredores e áreas de espera*				
None	Less than half	About half	More than half	Most	*Nenhuma*	*Menos que a metade*	*Metade*	*Mais que a metade*	*A maioria*
1	2	3	4	5	*1*	*2*	*3*	*4*	*5*
e. Telephone calls					*e. Ligações telefônicas*				
None	Less than half	About half	More than half	Most	*Nenhuma*	*Menos que a metade*	*Metade*	*Mais que a metade*	*A maioria*
1	2	3	4	5	*1*	*2*	*3*	*4*	*5*
f. Other (Please specify: ________________________)					*f. Outro (por favor, especifique: ________________)*				
None	Less than half	About half	More than half	Most	*Nenhuma*	*Menos que a metade*	*Metade*	*Mais que a metade*	*A maioria*
1	2	3	4	5	*1*	*2*	*3*	*4*	*5*
7. Now please think about all of the communication between staff and the family during the child's last 3 days. To the best of your knowledge, how much of that communication took place in:					*7. Agora por favor pense em toda a comunicação entre a equipe e a família durante os últimos 3 dias de vida da criança. Que você saiba, quanto dessa comunicação ocorreu em:*				
a. Family conferences					*a. Reuniões de família*				
None	Less than half	About half	More than half	Most	*Nenhuma*	*Menos que a metade*	*Metade*	*Mais que a metade*	*A maioria*
1	2	3	4	5	*1*	*2*	*3*	*4*	*5*
b. With the family during rounds					*b. Discussões com a família durante as visitas médicas*				
None	Less than half	About half	More than half	Most	*Nenhuma*	*Menos que a metade*	*Metade*	*Mais que a metade*	*A maioria*
1	2	3	4	5	*1*	*2*	*3*	*4*	*5*
c. Private conversations with the family at the bedside					*c. Conversas particulares com a família à beira do leito*				
None	Less than half	About half	More than half	Most	*Nenhuma*	*Menos que a metade*	*Metade*	*Mais que a metade*	*A maioria*
1	2	3	4	5	*1*	*2*	*3*	*4*	*5*
d. Non-private places, such as hallways and waiting areas					*d. Lugares não privados, tais como corredores e áreas de espera*				
None	Less than half	About half	More than half	Most	*Nenhuma*	*Menos que a metade*	*Metade*	*Mais que a metade*	*A maioria*
1	2	3	4	5	*1*	*2*	*3*	*4*	*5*
e. Telephone calls					*e. Ligações telefônicas*				
None	Less than half	About half	More than half	Most	*Nenhuma*	*Menos que a metade*	*Metade*	*Mais que a metade*	*A maioria*
1	2	3	4	5	*1*	*2*	*3*	*4*	*5*
f. Other (please specify: ________________________)					*f. Outro (por favor, especifique: ________________)*				
None	Less than half	About half	More than half	Most	*Nenhuma*	*Menos que a metade*	*Metade*	*Mais que a metade*	*A maioria*
1	2	3	4	5	*1*	*2*	*3*	*4*	*5*
8. How many family conferences did you participate in during:					*8. Quantas reuniões de família você participou durante:*				
a. The ICU stay in which the child died? (enter 0 if none)					*a. A internação na UTI na qual a criança morreu? (coloque 0 se nenhuma*				
Number of conferences: __________________				Don't know ☐	*Número de reuniões: __________________*				*Não sei* ☐
b. The last 3 days of the child's life (enter 0 if none)					*b. os últimos 3 dias de vida da criança (coloque 0 se nenhuma)*				
Number of conferences: __________________				Don't know ☐	*Número de reuniões: __________________*				*Não sei* ☐

Continue...

...continuation

ORIGINAL				BRAZILIAN VERSION			
9.How much informal conversation and contact did you have with the child's family during:				*9. Quanto contato e conversa informal você teve com a família da criança durante:*			
a. The ICU stay in which the child died?				*a. A internação na UTI onde a criança morreu?*			
None	Some	Quite a bit	A lot	*Nenhum*	*Pouco*	*Bastante*	*Muito*
1	2	3	4	*1*	*2*	*3*	*4*
b. The last 3 days of the child's life?				*b. Os 3 últimos dias de vida da criança?*			
None	Some	Quite a bit	A lot	*Nenhum*	*Pouco*	*Bastante*	*Muito*
1	2	3	4	*1*	*2*	*3*	*4*
10. Were you working at the time of the child's death?				*10. Você estava trabalhando no momento em que a criança morreu?*			
Yes		No		*Sim*		*Não*	
1		2		*1*		*2*	
11. Were you at the bedside at the moment death?				*11. Você estava à beira do leito no momento da morte?*			
Yes		No		*Sim*		*Não*	
1		2		*1*		*2*	
12. There are barriers to helping a patient and his/her family in the ICU. This section lists some barriers you may have encountered while working with this family. Please circle one answer for each item. The following was a barrier to providing care for this child and their family…				*12. Existem barreiras para ajudar um paciente e sua família na UTI. Esta seção lista algumas barreiras que você pode ter encontrado enquanto trabalhava com essa família. Por favor, circule uma resposta para cada item.*			
a. Not enough staff/heavy patient load				*a. Equipe insuficiente/volume grande de pacientes*			
Yes		No		*Sim*		*Não*	
1		2		*1*		*2*	
b. Patient too sick to allow interaction with family				*b. O paciente estava doente demais para permitir interação com a família*			
Yes		No		*Sim*		*Não*	
1		2		*1*		*2*	
c. Conflict with others on the team				*c. Conflito com membros da equipe*			
Yes		No		*Sim*		*Não*	
1		2		*1*		*2*	
d. Outside the scope of my practice				*d. Fora do alcance/realidade/rotina da minha prática*			
Yes		No		*Sim*		*Não*	
1		2		*1*		*2*	
e. Family did not visit or call				*e. A família não visitou ou telefonou*			
Yes		No		*Sim*		*Não*	
1		2		*1*		*2*	
f. Lack of communication among clinical staff				*f. Falta de comunicação entre a equipe clínica*			
Yes		No		*Sim*		*Não*	
1		2		*1*		*2*	
g. The family was angry				*g. A família estava zangada*			
Yes		No		*Sim*		*Não*	
1		2		*1*		*2*	
h. The family had unrealistic expectations of medical treatment				*h. A família tinha expectativas não realistas do tratamento médico*			
Yes		No		*Sim*		*Não*	
1		2		*1*		*2*	
i. Personal difficulty with this family				*i. Dificuldade pessoal com esta família*			
Yes		No		*Sim*		*Não*	
1		2		*1*		*2*	
j. Language difficulties				*j. Dificuldade com o idioma*			
Yes		No		*Sim*		*Não*	
1		2		*1*		*2*	
l. Other barriers?				*l . Outras barreiras?*			
Yes		No		*Sim*		*Não*	
1		2		*1*		*2*	
If yes, please specify: ______________________________				*Se sim, por favor, especifique: ______________________________*			
**SECTION C**				***SEÇÃO C***			
13. What do you wish could have been done differently for this child and family? Please explain.				*13. O que você desejaria que tivesse sido feito diferente para esta criança e família? Por favor, explique*			
14. What changes would you recommend in the way care near the end of life is provided? Please explain.				*14. Que mudanças você recomendaria na maneira que os cuidados perto do fim de vida são proporcionados? Por favor explique.*			
This completes the survey.<br>Please return it in the envelop provided.<br>THANK YOU VERY MUCH FOR YOUR HELP.				*Aqui termina a pesquisa.*<br>*Por favor, retorne-a no envelope fornecido.*<br>*MUITO OBRIGADO POR SUA AJUDA.*			
